# Vibrotactile enhancement in hand rehabilitation has a reinforcing effect on sensorimotor brain activities

**DOI:** 10.3389/fnins.2022.935827

**Published:** 2022-10-04

**Authors:** Qiang Du, Jingjing Luo, Qiying Cheng, Youhao Wang, Shijie Guo

**Affiliations:** ^1^Academy for Engineering and Technology, Fudan University, Shanghai, China; ^2^Shanghai Engineering Research Center of AI and Robotics, Shanghai, China; ^3^Engineering Research Center of AI and Robotics, Ministry of Education, Shanghai, China; ^4^Jihua Laboratory, Foshan, China; ^5^Department of the State Key Laboratory of Reliability and Intelligence of Electrical Equipment and the Hebei Key Laboratory of Robot Perception and Human-Robot Interaction, Hebei University of Technology, Tianjin, China

**Keywords:** vibrotactile stimulation, functional near-infrared spectroscopy, granger causal analysis, laterality index, hand rehabilitation training

## Abstract

**Objective:**

Stroke patients often suffer from hand dysfunction or loss of tactile perception, which in turn interferes with hand rehabilitation. Tactile-enhanced multi-sensory feedback rehabilitation is an approach worth considering, but its effectiveness has not been well studied. By using functional near-infrared spectroscopy (fNIRS) to analyze the causal activity patterns in the sensorimotor cortex, the present study aims to investigate the cortical hemodynamic effects of hand rehabilitation training when tactile stimulation is applied, and to provide a basis for rehabilitation program development.

**Methods:**

A vibrotactile enhanced pneumatically actuated hand rehabilitation device was tested on the less-preferred hand of 14 healthy right-handed subjects. The training tasks consisted of move hand and observe video (MO), move hand and vibration stimulation (MV), move hand, observe video, and vibration stimulation (MOV), and a contrast resting task. Region of interest (ROI), a laterality index (LI), and causal brain network analysis methods were used to explore the brain’s cortical blood flow response to a multi-sensory feedback rehabilitation task from multiple perspectives.

**Results:**

(1) A more pronounced contralateral activation in the right-brain region occurred under the MOV stimulation. Rehabilitation tasks containing vibrotactile enhancement (MV and MOV) had significantly more oxyhemoglobin than the MO task at 5 s after the task starts, indicating faster contralateral activation in sensorimotor brain regions. (2) Five significant lateralized channel connections were generated under the MV and MOV tasks (*p* < 0.05), one significant lateralized channel connection was generated by the MO task, and the Rest were not, showing that MV and MOV caused stronger lateralization activation. (3) We investigated all thresholds of granger causality (GC) resulting in consistent relative numbers of effect connections. MV elicited stronger causal interactions between the left and right cerebral hemispheres, and at the GC threshold of 0.4, there were 13 causal network connection pairs for MV, 7 for MO, and 9 for MOV.

**Conclusion:**

Vibrotactile cutaneous stimulation as a tactile enhancement can produce a stronger stimulation of the brain’s sensorimotor brain areas, promoting the establishment of neural pathways, and causing a richer effect between the left and right cerebral hemispheres. The combination of kinesthetic, vibrotactile, and visual stimulation can achieve a more prominent training efficiency from the perspective of functional cerebral hemodynamics.

## Introduction

Stroke is one of the leading causes of human death and disability worldwide, with typical sequelae of motor dysfunction, including loss of hand function (Katan and Luft, 2018). Due to the ability of the brain system to reorganize its structure and function, repetitive activation of brain areas through active and steady exercise and training can facilitate functional remodeling of the brain (Krebs et al., 1998). Pneumatic muscle-actuated soft rehabilitation gloves are now commonly used to help actuate the paralyzed hands in stroke patients for rehabilitation training (Bernocchi et al., 2018; Chu and Patterson, 2018). Studies have shown that subacute or chronic stroke patients may also lose part of their haptic perception (Wolfe, 2000). Pneumatic gloves alone do not provide adequate tactile input to the patient, making rehabilitation training somewhat limited (Heo et al., 2012; Carlsson et al., 2018). There is, therefore, a need to explore more effective forms of hand rehabilitation.

The principle of functional brain rehabilitation is to induce relevant activation of the cerebral cortex through the processing of external stimuli (Kaplan, 1988; Lin et al., 2013; Zheng et al., 2021). Studies have shown that hand haptic perception including both kinesthetic perception and tactile perception stimulate the somatosensory cortex (Li et al., 2021). Integration of kinesthetic and tactile stimuli for post-stroke rehabilitation can result in collective and prolonged improvement of motor control (Demain et al., 2013; Afzal et al., 2018; Seo et al., 2019). The kinesthetic stimulus can be delivered through a pneumatically actuated rehabilitation glove or hand exoskeleton devices that drive the affected limb and provide position, movement, and force information (proprioception) using muscle spindles, joint mechanoreceptors, and the Golgi tendon organs (Ballardini et al., 2018; Skorina et al., 2018). The tactile stimulus can be delivered through vibration, stretch or pressure at the affected limb and provide cutaneous information through mechanoreceptors to the central nervous system (Ranade et al., 2014; Seo et al., 2019). Mechanical stimulation is produced by vibration, as prominent and simple to administer, and the frequency of the delivered vibration can be modulated to convey information (Sherrick et al., 1990), which is shown to be useful to synthesize and deliver vibrotactile kinesthetic feedback to enhance stabilization and reaching actions performed with the arm and hand in neurotypical people (Krueger et al., 2017) and to improve proprioception (Cuppone et al., 2016). Studies have combined fingertip skin-tactile stimulation with exoskeleton-assisted hand rehabilitation to provide kinesthetic and tactile feedback to patients, improving training participation and promoting recovery of motor function in stroke patients (Li et al., 2021). A combined kinesthetic and tactile rehabilitation task can more effectively elicit the activation of the somatosensory cortex in brain areas (Breitwieser et al., 2012; Lim et al., 2015). The visual stimulation of hand movement observation can induce the excitation of mirror neurons in the brain, making the motor cortex area of the brain active (Pineda, 2008). Different types of sensory feedback stimulate the sensory-motor brain areas of the brain (Shokur et al., 2018). However, few studies have specifically investigated the effects of hybrid rehabilitation training combining kinesthetic, tactile, and visual. It is essential to investigate the effects of different combinations of sensory stimuli on cortical activation patterns to achieve more effective hand rehabilitation training.

The functional relationship between the two brain hemispheres during training in subjects is a key factor in promoting a better understanding of neuromotor control, which in turn can help improve rehabilitation strategies (Herold et al., 2017). Asymmetric processing of the sensory-motor cortex and differences between the left and right hemispheres have been commonly reported in many functional neuroimaging studies, and the phenomenon is referred to as hemispheric dominance or hemispheric asymmetry (Lin et al., 2013; Rea et al., 2014; Rahman et al., 2020; Rieke et al., 2020). Chieh-Ling used functional Near-Infrared Spectroscopy (fNIRS) to explore the cortical activation patterns associated with shoulder and finger movements in healthy adults (Yang et al., 2020). They found that bilateral cortical activation was prevalent in both motor tasks, with higher activation in the contralateral compared to the ipsilateral primary motor cortex (Yang et al., 2020). Laterality index (LI) was often used to quantify the phenomenon of hemispheric asymmetry and for understanding interhemispheric patterns of action and functional changes in the brain (Belfatto et al., 2018; Lemée et al., 2020; Guo et al., 2022). Delorme used the LI to the assess changes in the hemispheric balance between cortical sensorimotor areas in stroke patients (Delorme et al., 2019). Progressive lateralization observed over the 2 months evolved concomitantly with an increase in the Fugl-Meyer score (*p* < 0.001) (Delorme et al., 2019).

Effective connectivity is also used to explore the effects of different rehabilitation tasks on the network patterns of the brain and to discover underlying neural information (Rathee et al., 2016; Hu et al., 2020). Scholars Huo et al. (2019) used fNIRS imaging to study cortical activation and effective connectivity changes within functional brain networks after stroke with the limbic connectivity rehabilitation task. The results showed that effective connectivity was significantly lower in stroke patients than in healthy controls at rest and in the task state (*p* < 0.025) (Huo et al., 2019). Brain network analysis known as the causal networks can be used to analyze changes in the role of the brain between brain regions during a task state to clarify the causal connections between different regions of the brain and thus deeply explore brain hemispheric asymmetry (Abe et al., 2019; Chen et al., 2019; Tang et al., 2020). In EEG signals, the participation degree of task-related brain regions is different in three motor conditions (motor observation, motor imagery, and motor execution) (Zhu et al., 2022). With a lateralization advantage for both activation patterns of electroencephalography source imaging and causal network connections (Zhu et al., 2022). Zhao et al. (2016) used Granger causality (GC) analysis to explore the resting-state fMRI data between the ipsilesional M1 and the whole brain. The results suggested differences between left-hemisphere subcortical stroke and healthy human brain network connections (Zhao et al., 2016). Gao et al. (2011) found more effective causal connectivity circuits between selected seed regions during right-hand performance than during left-hand performance, demonstrating the effect of right-hand brain asymmetry on effective connectivity networks.

fNIRS is a critical technique for functional brain imaging with a high temporal resolution for detecting cortical hemodynamics. The technique is based on neurovascular coupling and can reflect the brain’s neural activity and hemodynamic changes in the corresponding brain regions during the task (Ferrari and Quaresima, 2012). Compared to functional Magnetic Resonance Imaging, fNIRS can be used independent of the environment, allowing easy and effective detection of physiological activity in the brain in a variety of experimental paradigms. Therefore, this experiment used fNIRS to investigate changes in brain activity during multiple sensory stimuli in the subject’s hand.

In this study, we investigate the hemodynamic working patterns of the brain’s sensorimotor under different combinations of stimulation rehabilitation tasks in terms of the fundamental physiological changes in the brain. We hypothesize that combining different stimulation modalities will result in a more potent contralateral activation LI effect of sensorimotor brain areas and a result in a more prosperous causal brain network of interactions between brain areas, allowing for more effective rehabilitation training. The mechanism of mixed stimulation rehabilitation training is clarified to provide a basis for designing more effective hand rehabilitation training programs.

## Materials and methods

### Experimental subjects

Fourteen participants volunteered for this project, including six males and eight females within the range of 24 ± 1.88 years old, all right-handed. The Ethical Committee of the Fudan University approved the study protocol, and the study was conducted in accordance with the principles of the Declaration of Helsinki regarding research involving human subjects. Subjects were healthy and free from neurological, psychiatric, or other brain-related disorders. Each subject was informed in detail of the purpose and precautions before the experiment and signed an informed consent form.

### Experimental setup

**FIGURE 1:**
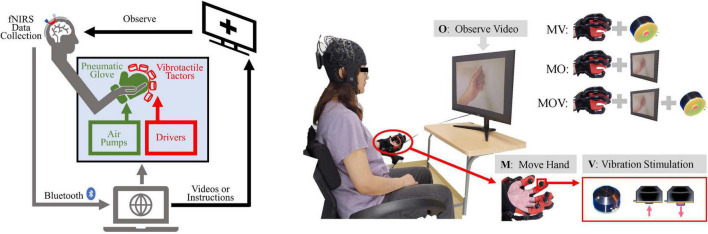
Schematic diagram of the experimental setup and paradigm. **(A)** Experimental system setup: left, functional near-infrared spectroscopy (fNIRS) data acquisition device; middle, vibrotactile enhanced pneumatically actuated hand rehabilitation device, consisting of a pneumatic glove rehabilitation module and a vibrotactile actuators; right, motion video and command display. Multiple devices were controlled synchronously using Python scripts and through USB serial communication. **(B)** The “O” represents observing the video, “M” represents moving the left hand with the pneumatic glove, and “V” represents vibrotactile stimulation to the abdomen of five fingertips. The tasks for the left hand consisted of Move Hand and Vibration Stimulation (MV), Move Hand and Observe Video (MO), Move Hand and Observe Video and Vibration Stimulation (MOV), and contrast task (Rest). The screen displays a cross when the experimental task did not include a video of the hand clenching motion.

The experimental system consisted of test subjects using a vibrotactile enhanced pneumatically actuated hand rehabilitation device and an fNIRS (Artinis Brite24TM from Artinis Medical Systems, Germany) acquisition device, which enables the communication between and control of multiple devices through lab-developed Python scripts. The experimental system can safely and effectively collect information on changes in cortical oxyhemoglobin (HbO) levels during rehabilitation training. The system schematic is shown in Figure 1A.

The tactile enhanced hand rehabilitation training device can deliver five-finger vibratory tactile stimulation during hand extension grip movement and increase the visual stimulation of the handgrip movement, as shown in the display screen in Figure 1B. The pneumatically actuated glove consists of a flexible pneumatic glove, control system, air pump drive system, and sound insulation system. This system allows for safe and effective pneumatic glove control to help the subject achieve a passive fist clenching and 180^°^ hand opening movement; it takes 2 s to complete hand flexion and extension movement (Bae et al., 2017).

The vibrotactile actuators are miniature electromagnetic solenoid-type stimulators (Dancer Design, St Helens, Merseyside, England) that are about in 18 mm diameter, 12 mm high, weigh 5.4 g, and have a maximum stimulation depth of 2 mm. The driving voltage is adjustable from 0 to 5 V, and the vibration frequency can be changed from 0 to 300 Hz. This experiment used a rated operating voltage of 5 V and a stimulation frequency of 100 Hz using square waves (Breitwieser et al., 2012). About 0.5 N was used for individual fingertip abdominal forces by tactile simulation. The vibrotactile actuators are fixed to the fingers by a specially made elastic fabric, and the red impact shaft of the actuators is close to the ventral side of the five fingertips.

### Experimental paradigm

**FIGURE 2:**
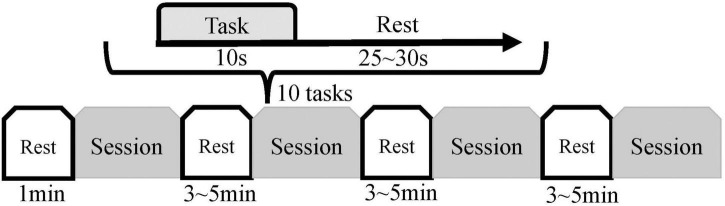
Experimental procedure. Each subject was introduced to the experimental procedure and tried out the equipment before the formal experiment. Each subject had four sessions, each consisting of 10 experimental tasks; the sequence of the task types within the 10 experimental tasks was randomized (Rest, MV, MO, and MOV). The duration of each task was 10 s, with a rest period of 25–30 s between tasks. Subjects performed a 1-min resting state before the experiment and rested for 3–5 min after each session.

Each subject sat in a chair and was encouraged to remain relaxed and comfortable, keeping the body still, with the left hand naturally resting on the left armrest of the chair. The subject’s left hand could be driven by the pneumatic gloves to passively perform the rehabilitation movements of fist-clenching and palm extension allowing the subject to feel the vibrotactile stimulation. The subject was allowed to observe on-screen instructions or a video of the hand clenching action. The above three stimulation modalities were combined to achieve three rehabilitation and control tasks, including Move Hand and Vibration Stimulation (MV), Move Hand and Observe Video (MO), Move Hand and Observe Video and Vibration Stimulation (MOV), and was also given time for a Rest mode, as illustrated on the top right corner of Figure 1B. The experimental procedure is shown in Figure 2. The experiment consisted of four sessions at intervals of 3–5 min. Each session comprised 10 given tasks. Four sessions for a total of 40 tasks. The task was 10 s, followed by a resting period randomly of 25–30 s. Ultimately each subject will complete the MV, MO, MOV, and Rest experimental tasks 10 times each.

### Data acquisition and preprocessing

**FIGURE 3:**
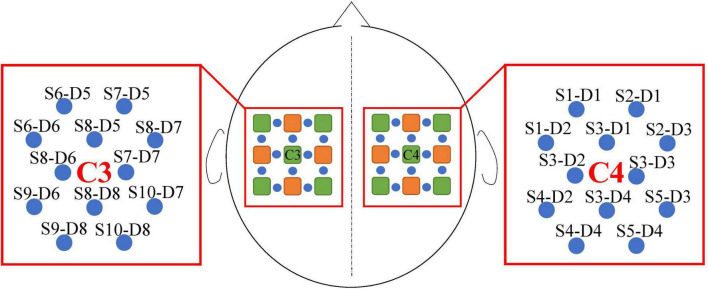
Channel distribution and designation. The fNIRS sources are green and fNIRS detectors are orange. The blue circles represent the fNIRS channels constructed by the source and detector, the names of which were S and D. In addition, S indicates the fNIRS source and D indicates the fNIRS detector.

We used a portable fNIRS acquisition to obtain the HbO data at a 10 Hz sampling rate during the experiments in this study. Each adjacent source–detector pair created one physiological fNIRS channel. Ten fNIRS sources and eight fNIRS detectors on the subject’s sensorimotor area resulted in 24 channels. The inter-optode distance was 3 cm. The data from the channels located around C3 and C4 of the international 10–20 systems were selected, resulting in 24 fNIRS channels (Beisteiner et al., 1995). Twelve of these channels were around channel C3 of the left hemisphere and 12 around channel C4 of the right hemisphere. The distribution of sources, detectors, and channels is shown in Figure 3.

Data were processed using the MNE/MNE-NIRS (Python 3.7) software package (Gramfort et al., 2013; Luke et al., 2020). Light intensity was converted into optical density and the HbO and deoxyhemoglobin (HbR) concentration information was calculated for the corresponding channels by a modified Beer-Lambert law. Physiological noises, such as breathing, heartbeat, and Mayer waves, are removed by a 0.02–0.2 Hz bandpass filter (Naseer and Hong, 2015; Pinti et al., 2019). A start of each task before 5 s was selected as the baseline range to adjust data for each task.

### Data analysis

#### Region of interest analysis

The HbO data for each channel in this experimental paradigm were analyzed. It has been shown that the averaging and GLM approach to fNIRS analysis provides similar response amplitude estimates at the group level (Luke et al., 2020). Also, averaging has a more intuitive interpretation. Therefore, the present study used superimposed to superimpose and averaged the hemodynamic responses of the brain in multiple identical task states. The effects of different rehabilitation tasks on cortical function were observed from a population perspective and changes in activation in sensorimotor brain regions were explored. The unpaired *t*-test function (Seabold and Perktold, 2010) was used to statistically test the amplitude of HbO under different rehabilitation tasks during the task time of interest (*p* = 0.05).

#### Cortical lateralization of response

Hand motor control is functionally lateralized in the brain. The left corresponds to the sensorimotor area of the cortex that controls the right hand, and the right corresponds to the sensorimotor area of the cortex that controls the left hand. To estimate the degree of asymmetry, we typically compute the LI using the fNIRS data (Jin et al., 2020; Matsuo et al., 2021). The major rationale for using the LI value is to facilitate the description of hemispheric dominance from functional activation patterns (Jin et al., 2020). Studies have shown that healthy people have more significant functional laterality than stroke patients (Takeda et al., 2014; Kruse et al., 2020). The effect of different rehabilitation tasks on the laterality of the brain can reflect the effect of rehabilitation training on the cerebral cortex. We quantified the phenomenon of functional laterality activation using an LI based on changes in the HbO content of the brain under different rehabilitation tasks. The LI algorithm was calculated as follows:


$$L\,I=f\times \frac{R\,H\,i-L\,H\,i}{\left|R\,H\,i\right|+\left|L\,H\,i\right|}$$

Where LI is the LI; *f* is the value of the adjustment factor (takes the value 1); RHi represents the mean HbO values for each channel in the right-brain region within 20 s of the task’s start; LHi represents the mean HbO left-brain region. The LI was calculated between the 12 channels in the left- and right-brain regions as homotopic connections, namely, anatomically symmetrical positional connections between the cerebral hemispheres, with a total of 12 channel connections.

When *f* is held to 1, the predefined laterality index threshold (LI_*TH*_) value is usually set to 0.2 (Jin et al., 2020). The LI value is -1 or 1 if activation happens exclusively in the left or right Region of **i**nterest (ROI), respectively. The LI value is near to zero if both ROIs are similarly activated, indicating no asymmetry (Matsuo et al., 2021). Cerebral hemisphere asymmetry is usually determined by the size of the LI compared to LI_*TH*_. Significant differences between laterality coefficients and thresholds in the population were calculated using a one-sample *t*-test function to observe statistically significant channels of laterality activation (*p* = 0.05) (Seabold and Perktold, 2010).

### Causal brain network analysis

During rehabilitation training, the brain acts as a functional network, and interactions between different regions of the brain occur. Using a Granger causal network, changes in the working state of the brain network under rehabilitation training were explored. Studies have shown that HbR contains more physiological interference components than HbO data (Homae et al., 2007). Therefore, this study used HbO data for calculation. To construct a stable causal brain network, we used the mean of HbO for each subject over multiple experimental tasks to calculate the causal network.

#### Granger causality analysis

GC is deemed suitable for multivariate time series data and requires no prior knowledge of the connectivity directions among different brain regions (Friston et al., 2013; Park and Friston, 2013). The GC test assumes that these variable time series include all the predictive information for both *y* and *x*. The following is an explicit description of GC using an autoregressive process, and the model order was identified using the Bayesian information criterion (Duggento et al., 2018; Du et al., 2022). The Durbin Watson Test was used to measure the autocorrelation of residuals in the regression analysis (Durbin and Watson, 1950; Duggento et al., 2018).


$$x_{t}=a_{0}+\sum_{i=1}^{p}a_{i}\,x_{t-i}+\sum_{i=1}^{q}b_{i}\,y_{t-i}+\varepsilon_{y\,x}\,\left(t\right)$$

$$x_{t}=a_{0}+\sum_{i=1}^{p}a_{i}\,x_{t-i}+\varepsilon_{x}\,\left(t\right)$$

We define the GC value of *y_t_* for *x_t_* to represent the magnitude of GC:


$$G\left(y\to x\right)=In\frac{C\,O\,V\,\left(\varepsilon_{x}\right)}{C\,O\,V\,\left(\varepsilon_{y\,x}\right)}$$

Similarly, the GC value of *x*_*t*_for *y_t_* can be defined:


$$G\left(x\to Y\right)=In\frac{C\,O\,V\,\left(\varepsilon_{y}\right)}{C\,O\,V\,\left(\varepsilon_{x\,y}\right)}$$

The higher the GC value, the more likely it is to increase. Conversely, if the value stays the same, the channel’s causality intensity is considered to decrease.

#### Constructing a causal network

**FIGURE 4:**
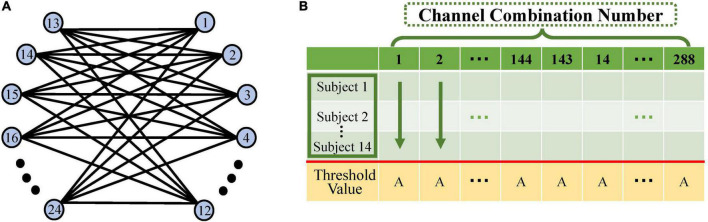
Schematic diagram of causal network computation. **(A)** The left–right hemisphere heterotopic connectivity model: blue circles indicate the 12 fNIRS channels on the left- and right-brain. Each channel in the left brain is connected to each in the right brain in both directions, resulting in 288 (12 × 12 × 2) channel combination pairs. **(B)** Schematic diagram of the calculation of significant channel pairs: horizontal coordinates indicate the 288 channel pairs in this table. Each column consists of the Granger causality (GC) values under a single task for 14 subjects. We calculated the significant difference between the 14 GC values and the threshold for each channel pair. Of the 288 channel pairs, those significantly greater than that threshold were screened out and used to construct the causal network.

A heterotopic connectivity model was constructed in this study to investigate the effect of the action between the left and right sensorimotor areas of the brain under different rehabilitation tasks (Li et al., 2015). Twelve channels in the left-brain region were connected to 12 in the right-brain region (Figure 4A). Due to the directional nature of the causal network model, 288 (12 × 12 × 2) combinations of connections were eventually formed and thus 288 causal values were obtained.

Significance was calculated using the one-sample *t*-test on the GC values of 14 subjects against the selected GC threshold (*p* = 0.05) (Seabold and Perktold, 2010). The channel pairs that were significantly larger than the threshold were screened out to construct a causal network (Figure 4B).

## Results

### Sensory stimuli cause contralateral hemodynamic activation of the cerebral cortex

**FIGURE 5:**
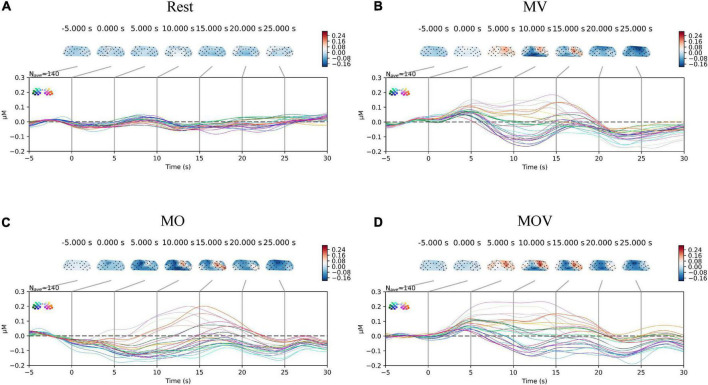
Change in cortical oxyhemoglobin (HbO) amplitude. The subplots **(A–D)** correspond to the changes in activation of the sensorimotor brain regions under the four task conditions of Rest, MV, MO, and MOV, respectively. The upper part of each subplot shows the changes in the topological HbO content. The topogram of the left part of the brain is the C3 brain region, and the right part is the C4 brain region. The different colors of the brain topogram correspond to the different values of HbO content, which correspond to the color bar on the right (unit is μMol/L). The lower part of each subplot shows the HbO data curve for each channel in μMol/L for a total of 24 channels. The data curves for each of the 24 channels are in μMol/L, with different colored data lines corresponding to different positions of the channels.

The combination of stimulations during rehabilitation task elicits the most prominent contralateral activation of brain regions. The HbO data were superimposed by overlaying HbO data from all subjections of multiple repetitive tasks (each rehabilitation task was generated by 14 subjects, each completing 10 tasks, for a total of 140 tasks). The mean HbO variation in the sensorimotor regions of the brain across the four task states was obtained. In Figure 5, differences in the changes in HbO at the left- and right-brain regions emerged between the three rehabilitation task conditions in brain regions MV, MO, and MOV. The left-brain region showed minor variation in activation, along with some degree of reverse activation, while the right-brain region showed more pronounced brain region activation at the same time. The left- and right-brain areas did not show significant brain area activation during the Rest periods (*p* > 0.05). The animation of the activation process of brain regions under Rest, MV, MO, MOV tasks can be seen in the Supplementary material.

**FIGURE 6:**
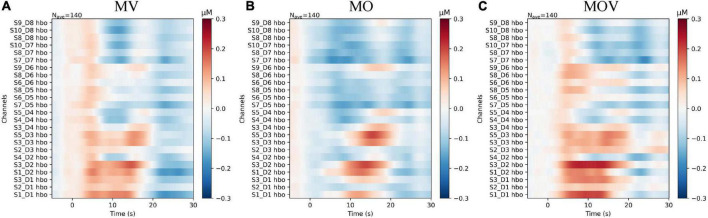
The subplots **(A–C)** correspond to the change in HbO content of each channel under the three rehabilitation tasks of MV, MO, and MOV, respectively. The horizontal axis of the subplot indicates the task time, with the task start time being 0 s and the task duration being 10 s. The vertical axis of the subplot indicates the distribution of channels, with the 12 left and right fNIRS channels in the upper and lower parts, respectively. The colors of the images correspond to the values of HbO, which ranged from −0.3 to 0.3 μMol/L.

To provide a more holistic view of the changes in cerebral HbO content under different rehabilitation tasks, we expressed the HbO of each channel for each task. Qualitative analysis revealed that HbO increased in multiple channels under each task. The range of activation in the right-brain region was greater than that in the left-brain region. The MV task caused activation in the right hemisphere, with no more pronounced activation manifested in the left hemispheres Figure 6A). The MOV task caused more activation range in the right hemisphere, with a more pronounced bias between the left and right hemispheres (Figure 6C). The MO task elicited a small range of activation in the right hemisphere, with no activation manifested in the left hemisphere (Figure 6B). The three tasks elicited different ranges of activation in the brain.

**FIGURE 7:**
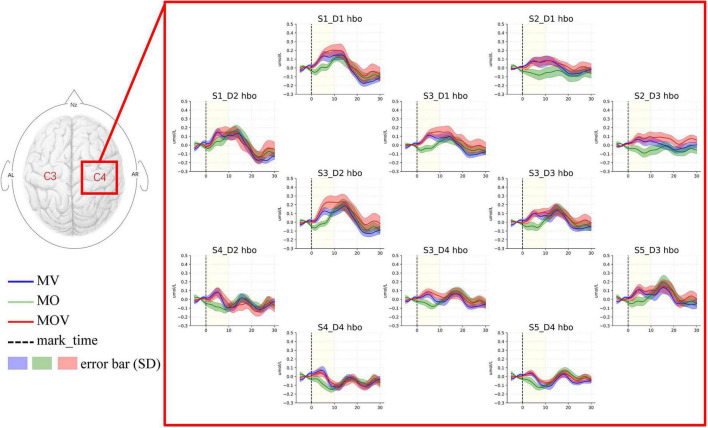
HbO topography of the right-brain sensorimotor. This figure shows the changes in HbO in the 12 fNIRS channels around C4 in the right-brain region under the three rehabilitation tasks. The task is performed from 0 s onward in each subplot, with the start moment indicated by the black dashed line. The task starts with a 5 s rest period before the start of the task. The task duration is the length of the 10 s time corresponding to the light-yellow shading. The blue, green, and red lines indicate the MV, MO, and MOV rehabilitation tasks. The different shaded areas are the error bar (HbO data for 14 subjects in that channel for that task. Standard deviation) corresponding to each line.

The left-brain region produced no significant activation under the three rehabilitation tasks of MV, MO, and MOV, the left-brain region produced no significant activation (*p* > 0.05), and the right-brain region produced a more significant activation (*p* < 0.05) (Figure 7). The HbO data from the 12 fNIRS channels in the right-brain region were mapped to brain region locations to observe the changes in HbO content in the sensorimotor brain region under the three rehabilitation tasks. The results show that at the 5 s of the rehabilitation task, the MV and MOV rehabilitation tasks elicited higher HbO amplitudes than those elicited by the MO rehabilitation task in multiple channels (*p* < 0.05; Table 1). Eight channels had significantly greater HbO amplitudes in MOV than in MO (S1_D1*, S3_D1^**^, S2_D3*, S3_D2^**^, S3_D3*, S4_D2*, and S3_D4*, S5_D4*). Eight channels (S2_D1*, S3_D1^**^, S2_D3*, S3_D2*, S3_D3*, S4_D2*, S3_D4*, and S4_D4*) also had significantly larger HbO amplitudes for MV than for MO, with no significant difference in HbO amplitudes between MOV and MV (*Indicates *p* < 0.05, ^**^ indicates *p* < 0.01). Thus, the results show that the MV rehabilitation tasks trigger brain area activation earlier during the task when compared with MOV rehabilitation tasks.

**TABLE 1:** Differences in channel cortical oxyhemoglobin content amplitude between move hand and vibration stimulation (MV), move hand and observe video (MO), move hand and observe video and vibration stimulation (MOV) tasks at 5 s of the recovery task.

Channel name	MOV > MO (*p*-value)	MOV > MV (*p*-value)	MV > MO (*p*-value)
S1_D1	0.017*	0.715	0.061
S2_D1	0.054	0.918	0.033*
S1_D2	0.191	0.873	0.093
S3_D1	0.004**	0.749	0.003**
S2_D3	0.030*	0.688	0.043*
S3_D2	0.004**	0.348	0.012*
S3_D3	0.027*	0.995	0.013*
S4_D2	0.049*	0.912	0.023*
S3_D4	0.012*	0.605	0.037*
S5_D3	0.074	0.859	0.051
S4_D4	0.050	0.580	0.025*
S5_D4	0.027*	0.611	0.056

* and ** indicate statistical significance at the *p* < 0.05 and *p* < 0.01 levels, respectively.

### Stronger cortical lateralization in tasks containing vibrotactile enhancement

**FIGURE 8:**
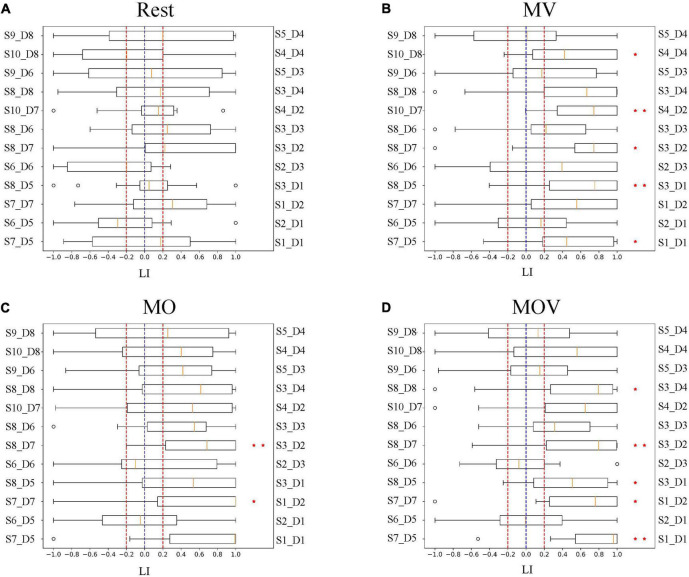
Cortical lateralization. The subplots **(A–D)** show the distribution of activation laterality index (LI) for each pair of channel groups under the four task conditions, Rest, MV, MO, and MOV. As shown, the horizontal coordinates of the subplots are the LI, with values ranging from −1 to 1. The vertical axis indicates the symmetrically located channel groups for 12 channels. Box plots are used to represent the distribution of the LI for 14 subjects under that channel group for that rehabilitation task. They are compared to the threshold of 0.2 for the significance LI at the group level (* indicates *p* < 0.05, ** indicates *p* < 0.01).

To quantify the lateralized activation of brain area function under different rehabilitation tasks, we used the LI for characterization. The results show that the brain regions showed lateralized activation under the MV, MO, and MOV rehabilitation tasks (Figure 8). The MV produced five significant lateralized channel connections (S10_D8 and S4_D4*, S10_D7 and S4_D2^**^, S8_D7 and S3_D2*, S8_D5 and S3_D1^**^, and S7_D5 and S1_D1*). The MOV also produced five significate lateralized channel connections (S8_D8 and S3_D4*, S8_D7 and S3_D2^**^, S8_D5 and S3_D1*, S7_D7 and S1_D2*, and S7_D5 and S1_D1^**^). These selected channel pairs had significantly larger activation LI compared to the threshold value (Figures 8B,D). Meanwhile, the MO produced two (S8_D7 and S3_D2^**^, and S7_D7 and S1_D2*). In contrast, Rest had no lateralized channels.

### Combining tactile and kinesthetic elicit richer causal networks

**FIGURE 9:**
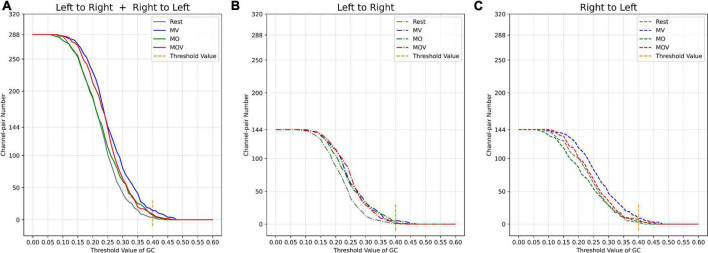
Relationship between threshold selection and the number of channel pairs. **(A)** The number of causal connections from the left- to the right brain and from the right- to the left brain by different GC thresholds. **(B)** The number of causal connections from the left- to right brain by different GC thresholds; **(C)** the number of causal connections from the right- to the left brain by different GC thresholds.

The brain has different working effects in brain regions under Rest, MV, MO, and MOV. As shown in Figure 9A, the combined tactile and kinesthetic MV elicited a more prosperous causal network when using a GC threshold from 0.15 to 0.45. The number of channel pairs under the MV was greater than that under the Rest, MO, and MOV. At GC thresholds of 0.15–0.25, MV and MOV that included tactile stimuli elicited richer causal networks. At thresholds of 0.3–0.4, MO and MOV containing visual stimuli elicited a more similar number of enriched causal networks. Figure 9B shows a plot of the number of causal connections from left- to right-brain areas, with the lowest number of causal connections in the Rest state and more similar performance in the other training tasks. Figure 9C, with GC thresholds selected from 0.15 to 0.45, showed that the MV elicited the highest number of causal connections from right to left-brain areas when compared with Rest, MO, and MOV training tasks.

The channel pair number of causal networks differed when using different GC thresholds. Choosing a suitable threshold can allow the researcher to observe the brain network properties under different tasks more effectively. According to the range of the number of causal connections (Figures 9A–C), when the GC threshold value was 0.4, the number of causal connections ranged from 5 to 13, which can allow researchers to better observe the differences of the brain causal networks under different rehabilitation tasks. At the same time, a larger GC value can fully reflect the causal characteristics between different channels.

**FIGURE 10:**
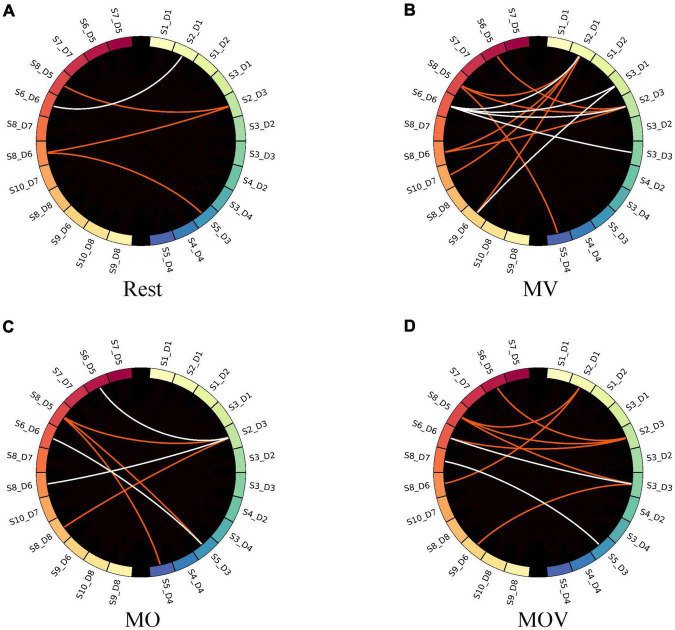
Causal network visualized. The subplots **(A–D)** represent the brain causal network maps under the four tasks, Rest, MV, MO, and MOV. The left and right halves of each subplot represent the 12 fNIRS channels in the left- and right-brain regions, respectively. The red and white colors represent the flow of information from the right to left brain and the left brain to right brain, respectively.

When the GC threshold was 0.4, the patterns of the causal network under the four rehabilitation tasks varied. As shown in Figure 10, the MV task containing kinesthetic and tactile sensations produced a richer causal brain network between the left- and right-brain regions, with S2_D1 and S2_D3 having a stronger causal relationship effect on the left-brain region (Figure 10B). With the left hand at rest in the rehabilitation glove device, only four causal networks were connected in the brain network (Figure 10A). Under the MO and MOV, the brain regions were less rich in causal networks, containing seven and nine causal connections, respectively (Figures 10C,D). However, MV showed a major causal network of connectivity patterns flowing from the right to the left (Figure 10D).

## Discussion

This study investigated the effects of tactile-enhanced hand training on the brain by analyzing HbO and interhemispheric cortical activity patterns in the brain’s left and right sensorimotor brain regions. Fourteen healthy subjects were recruited for the study, and the subjects’ less-preferred hand was trained with Rest, MV, MO, and MOV tasks. A qualitative analysis of HbO in sensorimotor brain regions revealed that kinesthetic, tactile, and visual combinations can cause the most extensive cerebral blood flow activation in the brain. LI analysis found strong cortical lateralization under a rehabilitation task containing tactile stimuli. Using Granger causal network analysis, it was found that the combination of tactile and kinesthetic stimuli can elicit stronger interhemispheric effects and promote the formation of new neural pathways. According to our findings, vibrotactile stimulation as a tactile enhancement modality can help trainers to generate stronger tactile input during training tasks, achieve stronger stimulation of the brain’s sensorimotor brain areas, and avoid poor training outcomes caused by reduced tactile sensory function. Therefore, tactile enhancement of hand rehabilitation is expected to achieve more effective rehabilitation results.

Combinations of stimulations during hand rehabilitation tasks can cause the most prominent cerebral blood flow activation. Training methods that effectively induce activation of brain regions are a crucial part of hand motor rehabilitation, and in clinical practice, passive hand movements are often used as external stimuli to induce brain activation in stroke patients with severe hand motor dysfunction. The brain achieves remodeling of motor function during repeated activation (Krebs et al., 1998). A variety of exoskeleton-type or pneumatically actuated hand rehabilitation robots has been created for hand rehabilitation training. Researchers analyzed the brain activation of passive movements and found that the execution of a rehabilitation robot hand can induce activation of the contralateral cortex mainly in the primary sensorimotor cortex, premotor cortex, and supplementary motor area (Szameitat et al., 2012; Chang et al., 2014; Lee et al., 2018). This study found that a combination of kinesthetic, tactile, and visual induced stronger activation in the sensorimotor regions of the brain than a single stimulus modality. Qualitative analysis of Figures 5, 6 shows that all three training tasks, MV, MO, and MOV, caused activation in sensorimotor areas of the brain. With MOV, the activation range was greater than the MV activation range, the activation range of MV was greater than that of MO, and Rest do not cause brain area activation (Figures 5A–D). The HbO content of multiple channels increased under the rehabilitation task, with the greatest activation intensity in brain regions for MOV and greater activation intensity for MV than for MO (Figures 6A–C). The rehabilitation tasks containing tactile stimuli (MV and MOV) elicited more rapid functional activation in the right brain. As shown in Figure 7, the HbO amplitude under the MV and MOV tasks was significantly greater than that induced by the MO task at 5 s. By comparison, we found that vibrotactile stimulation induced a more rapid activation in its contralateral brain region when compared with visual stimulation. The above phenomena suggest that MOV mixed stimulation can achieve brain function activation more effectively than other methods studied here and promotes cortical function recovery. In the MOV rehabilitation task, the cerebral blood flow response was enhanced but did not show a superimposed effect in the mixed kinesthetic, tactile, and visual situations.

Rehabilitation tasks that include tactile stimulation can induce stronger lateralized activation. Stimulation of an affected limb of a stroke patient can cause simultaneous activation of the left- and right-brain regions in the task state. The function of the damaged brain area is compensated for by other brain areas so that the activation of the left- and right-brain areas appears to be correlated, making hemispheric dominance diminished. Effective rehabilitation can take advantage of the functional plasticity of the brain to establish new neural pathways that allow other brain regions to compensate for the diminished function of the damaged brain regions and the body regains interhemispheric functional asymmetry. The brain gradually shifts from early bilateral to unilateral activation as the rehabilitation is effective (Delorme et al., 2019). Therefore, investigating the degree of lateralized brain activation under different rehabilitation tasks can help to clarify the effect of rehabilitation training. Some scholars find that tactile stimuli can cause lateral activation in the brain, such as tactile vibrations of the fingers and electrical stimuli that cause lateral activation (Han et al., 2018; Jin et al., 2020). Other studies have found that hand rehabilitation exercises that include tactile sensation can help patients improve their training attention and rehabilitation outcomes (Li et al., 2021). In this study, we analyze the fundamental physiological changes in the brain under vibrotactile enhanced hand training. The LI quantified the interhemispheric functional asymmetry under different rehabilitation tasks. The contrast task, Rest, did not induce lateral activation. The rehabilitation training of the MO task produced two significantly lateralized channel pairs. Both MV and MOV produced five significantly lateralized channel pairs for the two rehabilitation training tasks containing vibrotactile enhancement (Figure 8). Thus, vibrotactile enhanced-based hand rehabilitation can produce stronger lateralized activation of brain regions and facilitate the restoration of hemispheric functional dominance in the brain.

The combination of kinesthetic and tactile stimuli can elicit a richer causal brain network between hemispheres. Studies have shown that the action between brain regions contains more information about the underlying physiological brain activity. The effects of brain activity between brain regions in different rehabilitation tasks can be more effectively explored using brain network analysis. Some scholars have found that brain network changes are generated between brain regions under different types of experimental paradigm stimulation. For example, different causal networks appear between the C3 and C4 regions of the brain during left- and right-hand motor imagery (Hu et al., 2014; Chen et al., 2019). Motor execution also induces changes in the flow of information between brain regions (Youssofzadeh et al., 2014; Wang et al., 2016). The present study found that hand training with vibrotactile enhancement elicited a richer causal network. Figure 9 shows the performance characteristics of the causal network change across training tasks during the change of GC thresholds. Choosing different computational thresholds, the MV rehabilitation training task with a combination of kinesthetic and tactile stimuli produced richer causal networks when compared with the MO and MOV rehabilitation tasks. The left hand produced fewer causal connections under the strapped rehabilitation device form in the Rest state. Hand training tasks with vibrotactile enhancement effectively facilitate interactions between the left and right sensorimotor of the brain and promote the formation of new neural pathways. The MV task has a richer causal network than the MO task. The inclusion of vision influenced the allocation of resources in the brain, which influenced sensorimotor areas. With the change of the GC threshold, the number of causal connections flowing from the right to the left was the highest under the MV rehabilitation task. The combination of tactile and kinesthetic sensations could effectively promote specific flow changes between brain regions, i.e., the right-brain region’s more substantial causal effect on the left-brain region. This information flow is consistent with the direction generated by left-hand motor imagery and motor execution.

The paradigm of tactile enhancement can build a more effective brain–computer interface (BCI) with neurological feedback. The use of a BCI is a promising field in future neurological rehabilitation modalities that decodes the patient’s motor intent, triggering a rehabilitation device to complete a closed-loop rehabilitation exercise. Currently, neurofeedback often uses video feedback or rehabilitation devices. A single feedback modality does not produce effective neurofeedback for the patient. The tactile stimulation enhanced as the feedback paradigm for a BCI can effectively stimulate the cortical sensorimotor areas, strengthen neural pathways, promote neural remodeling in the brain, and is potentially valuable in improving the outcome of rehabilitation training for stroke patients.

This paper still has significant shortcomings, such as the lack of an in-depth investigation of the mechanism of the tactile enhancement effect, a lack of actual stroke patient data, and the failure to quantify the enhancement of the effect. Further research will, therefore, be necessary to explore the patterns and selection methods of stimulation patterns and intensities in stroke patients. In the future, the use of a tactile enhancement paradigm will be considered as a neurofeedback modality for BCI rehabilitation training to achieve more effective hand rehabilitation training. In addition, EEG and fNIRS data acquisition systems will also be used to detect fundamental physiological activity changes in the brain during rehabilitation training and build a stable and effective motor rehabilitation assessment system for stroke patients.

## Conclusion

In this paper, we constructed a vibrotactile and enhanced pneumatically actuated hand rehabilitation device. From the perspective of functional cerebral hemodynamics, we validate the effectiveness of the training tasks by ROI, LI, and causal network methods from multiple perspectives. We found that kinesthetic, tactile, and visual combinations can elicit broader contralateral activation in the brain. Training tasks containing vibrotactile enhancement elicit faster contralateral activation of sensorimotor brain areas and stronger cortical lateralization while eliciting a richer causal network in the brain. This study demonstrates that vibrotactile stimulation is effective in activating sensorimotor cortical of the brain and promoting interactions between brain areas, which facilitates the restoration and construction of cortical neural pathways, providing theoretical support for the effectiveness of vibrotactile enhanced hand rehabilitation training.

## Data availability statement

The raw data supporting the conclusions of this article will be made available by the authors, without undue reservation.

## Ethics statement

The studies involving human participants were reviewed and approved by the Ethical Committee of the Fudan University. The patients/participants provided their written informed consent to participate in this study.

## Author contributions

QD performed the research, analyzed the data, and drafted the article. JL and SG designed the research, supervised the project, and revised the article. QD, QC, and YW collected and interpreted the data. All authors reviewed the manuscript and agreed to be accountable for the content of the work.

## Supplementary material

Supplementary Video 1 Activation animation of brain areas. The subplots **(A–D)** represent activation animation of brain areas under MV, MOV, MO, and Rest tasks. The duration of task was 10 s. The topogram of the left part of the brain is the C3 brain region, and the right part is the C4 brain region. The different colors of the brain topogram correspond to the different values of HbO content, which correspond to the color bar on the right (unit is μMol/L).
